# A phase I, dose-finding study of sunitinib in combination with irinotecan in patients with advanced solid tumours

**DOI:** 10.1038/sj.bjc.6605852

**Published:** 2010-08-17

**Authors:** E Boven, C Massard, J P Armand, C Tillier, V Hartog, N M Brega, A M Countouriotis, A Ruiz-Garcia, J C Soria

**Affiliations:** 1 Department of Medical Oncology, VU University Medical Center, De Boelelaan 1117, Amsterdam NL-1081 HV, The Netherlands; 2 Department of Medicine, Institut Gustave Roussy, 39 Bis, Rue Camille Desmoulins, Villejuif 94800, France; 3 Pfizer Italia Srl, Torre A, via Lorenteggio 257, Milan 20152, Italy; 4 Pfizer Oncology, 10646 Science Center Drive, San Diego, CA 92121, USA

**Keywords:** sunitinib, irinotecan, combination, advanced solid tumours, pharmacokinetics

## Abstract

**Background:**

Sunitinib is a multitargeted, oral tyrosine kinase inhibitor with antitumour and antiangiogenic activity. We investigated the safety and pharmacokinetics of sunitinib in combination with irinotecan in patients with advanced, refractory solid tumours.

**Methods:**

Sunitinib was initially administered once daily at 37.5 mg per day on days 1–14 of a 21-day cycle, in which irinotecan 250 mg m^−2^ was given on day 1. In a second cohort, the sunitinib dose was reduced to 25 mg per day. Blood samples were collected for pharmacokinetic studies.

**Results:**

In the sunitinib 37.5 mg per day cohort, 3 out of 10 evaluable patients had objective responses, but dose-limiting toxicities (DLTs) of neutropenia, pneumococcal sepsis, and fatigue were observed. There were no DLTs in the sunitinib 25 mg per day cohort. Paired observations of pharmacokinetic parameter values of sunitinib and irinotecan alone *vs* the combination did not reveal significant drug–drug interactions. The maximum tolerated dose was defined as sunitinib 25 mg per day (days 1–14) with irinotecan 250 mg m^−2^ (day 1), but no activity was observed at this dose.

**Conclusion:**

Although a higher sunitinib dose of 37.5 mg per day (days 1–14) with irinotecan showed preliminary evidence of antitumour activity, this dose was poorly tolerated. Therefore, this particular combination will not be pursued for further studies.

The addition of targeted agents to standard chemotherapy is becoming widely investigated in a number of advanced solid tumour types, and has met with success in some malignancies, including colorectal cancer (CRC). The combination of the vascular endothelial growth factor (VEGF)-targeted monoclonal antibody bevacizumab with either irinotecan/5-fluorouracil (5-FU)/leucovorin (LV) (FOLFIRI) or oxaliplatin/5-FU/LV (FOLFOX) has shown increased median progression-free survival and/or overall survival in patients with advanced CRC as compared with chemotherapy alone (Hurwitz *et al*, 2004, 2005; Giantonio *et al*, 2007). The optimal role of targeted agents, sequencing of therapies, and primary tumour resistance as well as resistance development are under investigation in advanced CRC (Gravalos *et al*, 2007), as more effective treatment regimens are still needed.

Sunitinib is an oral tyrosine kinase inhibitor that is known to block the signalling activity of VEGF receptors (VEGFR-1, -2, and -3), platelet-derived growth factor receptors (-*α* and -*β*), stem-cell factor receptor (KIT), FMS-like tyrosine kinase 3, colony-stimulating factor-1 receptor, and rearranged during transfection (RET) ligand; glial cell line-derived neurotrophic factor receptor (Abrams *et al*, 2003; Mendel *et al*, 2003; Murray *et al*, 2003; O'Farrell *et al*, 2003; Kim *et al*, 2006). It is approved multinationally for the treatment of advanced renal cell cancer and imatinib-resistant/-intolerant gastrointestinal stromal tumours (Goodman *et al*, 2007). The tolerability profile of sunitinib is well established, and adverse events (mainly diarrhoea, mucositis, skin abnormalities, and altered taste) are generally manageable and reversible (Demetri *et al*, 2006; Motzer *et al*, 2007). In addition, hypertension and fatigue have been reported with sunitinib and are, in general, common side effects of VEGF pathway-targeted therapies (Demetri *et al*, 2006; Hutson *et al*, 2008; Launay-Vacher and Deray, 2009). Promising single-agent antitumour activity across patients with a range of solid tumour types has been observed in phase I and II trials of sunitinib, including neuroendocrine tumours, breast cancer, hepatocellular cancer, and non-small-cell lung cancer (NSCLC; Burstein *et al*, 2008; Kulke *et al*, 2008; Socinski *et al*, 2008; Faivre *et al*, 2009). Single-agent sunitinib has also shown modest antitumour activity in patients with metastatic CRC refractory to standard chemotherapy in a phase II trial (Saltz *et al*, 2007), suggesting that a study of sunitinib in combination with standard chemotherapy for metastatic CRC is warranted.

The established topoisomerase I inhibitor irinotecan (CPT-11), a derivative of the natural alkaloid camptothecin, prevents repair of single-strand breaks in DNA, resulting in double-strand DNA damage and cell death. Irinotecan is approved for treatment of advanced/metastatic CRC. The drug has also demonstrated antitumor activity in glioblastoma and both small-cell and NSCLC (Fukuoka *et al*, 1992; Masuda *et al*, 1992; Friedman *et al*, 1999). The most common grade 3 or 4 adverse events observed in a phase III trial of single-agent irinotecan administered every 3 weeks were neutropenia, diarrhoea, and vomiting (Fuchs *et al*, 2003).

The activity of sunitinib and irinotecan, together with their manageable and generally nonoverlapping toxicity profiles, suggests that combining the two agents may be beneficial in a broad range of solid tumours, particularly CRC. Here, we report results from a phase I, dose-finding study of sunitinib and irinotecan in patients with advanced solid tumours and included plasma pharmacokinetics assessed for the drugs alone and combined.

## Patients and methods

### Study design and treatment regimen

Sunitinib and irinotecan were administered in 3-week cycles, up to a maximum of 12 cycles. Seven days before the start of cycle 1, a single day's dose of sunitinib was administered to allow for the collection of samples for pharmacokinetic analysis. Patients subsequently received sunitinib as once-daily oral doses on days 1–14 followed by a 1-week break, and irinotecan as a 1-h intravenous infusion on day 1.

For combination of sunitinib and irinotecan, starting doses were selected being 75% of single-agent doses, that is, sunitinib 37.5 mg (50 mg daily oral dose as single agent) and irinotecan 250 mg m^−2^ (350 mg m^−2^ 3-weekly i.v. dose as single agent). The study design allowed for sunitinib a dose escalation to 50 mg and de-escalation to 25 mg, whereas the initial dose of irinotecan could be escalated to 300 or 350 mg m^−2^. Concomitant medication precluded, among others, potent CYP3A4 inhibitors and inducers. In addition, prophylactic use of hematopoietic growth factors to support neutrophil or platelet counts was not recommended in cycle 1, but could be used in subsequent cycles.

Dose-limiting toxicity (DLT) was defined as any of the following events occurring during the first 2 cycles of treatment that were attributable to the study drug combination: grade 4 neutropenia lasting ⩾7 days; febrile neutropenia (grade 3 or 4 neutropenia and fever ⩾38.5°C); neutropenic infection (grade 3 or 4 neutropenia with ⩾grade 3 infection); either grade ⩾3 thrombocytopenia with bleeding, or grade 4 thrombocytopenia lasting ⩾7 days; grade 3 or 4 nonhaematological toxicities including fatigue lasting ⩾7 days (except for skin or hair discolouration, alopecia, hyperamylasaemia, or hyperlipasaemia without other clinical evidence of pancreatitis, and asymptomatic hyperuricaemia; nausea, vomiting, or diarrhoea had to persist at grade 3 or 4 despite maximal medical therapy to qualify for DLT). MTD was the primary endpoint of the study, defined as the dose level at which none or one out of six patients experienced a DLT, with the next higher dose level having at least two out of three or two out of six patients encountering DLT during the first 2 cycles of combination therapy.

The study was conducted with institutional review board/independent ethics committee approval and in accordance with the International Conference on Harmonisation Good Clinical Practice guidelines, as well as applicable local laws and regulatory requirements. All patients provided written informed consent.

### Patient eligibility

Patients eligible for the study had histologically or cytologically proven advanced malignancy refractory to standard therapy, or for which no curative therapy was available, and were suitable for treatment with irinotecan; Eastern Cooperative Oncology Group performance status of 0 or 1, and a life expectancy ⩾12 weeks. Patients were excluded if they had received chemotherapy, radiation therapy, surgery, or investigational agent within 4 weeks before study entry, or previous irradiation to >25% of the bone marrow, grade ⩾2 neuropathy (any cause), uncontrolled brain metastases, myocardial infarction, severe/unstable angina, coronary/peripheral artery bypass graft, congestive heart failure, cerebrovascular accident including transient ischaemic attack, or pulmonary embolus within the 12 months before starting study treatment. Patients with uncontrollable hypertension (>150/100 mm Hg), grade 3 haemorrhage <4 weeks before starting study treatment, cardiac dysrhythmias (grade ⩾2), atrial fibrillation, QTc interval >450 ms (males) or >470 ms (females), or a history of grade 3 or 4 toxicity or severe hypersensitivity reaction associated with previous irinotecan treatment were also excluded.

### Patient assessments

The number of patients to be enroled was to be determined by the observed safety profile, which also determined the number of patients per dose level and the number of dose escalations. All patients who received at least one dose of study medication were included in the study analyses.

Patients underwent regular physical examinations (usually on day 1 of each treatment cycle), laboratory tests (blood, usually on days 1 and 15 of each cycle, and urinalysis) and 12-lead electrocardiogram (at screening, at steady state level of sunitinib on cycle 2 day 1, and as clinically indicated in subsequent cycles). Adverse events were graded according to the National Cancer Institute Common Terminology Criteria for Adverse Events (NCI CTCAE), version 3.0 (http://ctep.cancer.gov/protocoldevelopment/electronic-applications/docs/ctcaev3.pdf). Tumour measurements were assessed at screening, at every third cycle of chemotherapy, and when disease progression was suspected. Response Evaluation Criteria for Solid Tumors version 1.0 (Therasse *et al* 2000) was used, requiring repeat imaging studies ⩾4 weeks after the initial documentation of response.

### Pharmacokinetics

Pharmacokinetic analysis was carried out in a central laboratory, and pharmacokinetic parameter values were estimated using noncompartmental methods. Human potassium EDTA plasma pharmacokinetic samples were analysed for sunitinib and SU012662 and total drug (sunitinib plus SU012662) concentrations at BASi (West Lafayette, IN, USA) with the use of a validated, sensitive, and specific liquid chromatographic-tandem mass spectrometric method. The performance of the method during validation has been documented in the method validation report (BASi report 1000-05793-1). Plasma specimens were stored at −20°C until assay, and all samples were analysed within the 377 days of established stability. Calibration standard responses met acceptance criteria over the range of 0.100 to 60.0 ng ml^−1^ for sunitinib and 0.100 to 20.0 ng ml^−1^ for SU012662 using a quadratic regression with 1/concentration^2^ weighting. The lower limit of quantitation (LLOQ) for both sunitinib and SU012662 was 0.100 ng ml^−1^. The between-day assay accuracy, expressed as the ratio (%) of the estimated to the theoretical quality control (QC) concentrations, ranged from −1.1 to 1.3% for the low, medium, and high sunitinib QCs and from −1.0 to 3.6% for the low, medium, and high SU012662 QCs. Assay precision, expressed as the between-day coefficients of variation (%) of the estimated concentrations of QC samples, was ⩽6.5% for the low, medium, and high sunitinib QCs and ⩽6.9% for the low, medium, and high SU012662 QCs.

Human sodium heparin plasma pharmacokinetic samples were analysed for irinotecan and SN-38 concentrations at Eurofins AvTech Laboratories (Portage, MI, USA) using a validated, sensitive, and specific high performance liquid chromatographic method with fluorescence detection. The performance of the method during validation has been documented in the method validation report (Eurofins AvTech Laboratories: report 94-455.07). The plasma specimens were stored at −20°C until assay, and all samples were assayed within the 1860 days (for irinotecan) and 1020 days (for SN-38) of established stability. Calibration standard responses met acceptance criteria over the range of 1.28 to 3840 ng ml^−1^ for irinotecan and 0.480 to 640 ng ml^−1^ for SN-38, using a linear regression with 1/concentration weighting. The LLOQ was 1.28 ng ml^−1^ for irinotecan and 0.480 ng ml^−1^ for SN-38. The between-day assay accuracy ranged from −0.7 to 3.8% for the low, medium, and high irinotecan QCs and from −8.0 to 3.2% for the low, medium, and high SN-38 QCs. Assay precision was ⩽6.3% for the low, medium, and high irinotecan QCs and ⩽7.1% for the low, medium, and high SN-38 QCs.

Sunitinib was assessed on day −7 (i.e., 1 week before cycle 1); samples were taken pre-dose and at 1, 2, 4, 6, 8, 10, and 24 h post-dose. Irinotecan was evaluated on cycle 1 day 1; samples were taken before infusion and at 1, 1.5, 2, 4, 6, 8, 10, and 24 h after the start of treatment. In cycle 1, sunitinib administration started on day 2. Both agents were evaluated in combination on cycle 2 day 1; samples were drawn before drug administration and at 1, 1.5, 2, 4, 6, 8, 10, and 24 hours post-dose.

Pharmacokinetic parameter values were calculated for each subject by noncompartmental analysis of concentration–time data using WinNonlin version 4.1 (http://pharmacy.ucsf.edu/irc/pdfs/wul_users_guide.pdf). Actual sample collection time was used for sunitinib, SU012662, total drug, irinotecan, and SN-38. If pre-dose concentration for an individual was >5% of *C*_max_, a carryover correction was made as recommended by FDA guidance (FDA, 2003). Summary descriptives of pharmacokinetic values were presented only for paired observations with respect to each analyte. In the case where the dose for one of the paired observations was different from the other observation, dose correction to the intended dose was performed (correction factor: intended dose/actual dose). Dose correction to the MTD was performed. Individual patient trough plasma concentrations were summarised per cycle and study day. All concentrations that were below the limits of quantitation (BLQ) were set to zero before computation of descriptive statistics (BLQ values were excluded from the calculations of geometric means and the associated 95% confidence intervals).

Standard plasma pharmacokinetic parameters were used including the maximum plasma concentration (*C*_max_), time to *C*_max_ (*T*_max_), area under the plasma concentration–time curve to the time of the last measurable concentration (AUC_0–last_) or infinity (AUC_0–∞_), clearance, and terminal elimination half-life (t_1/2_). The geometric mean ratio was used to give a robust measurement of differences in exposure in rate (*C*_max_) and extent (AUC), with values below 0.8 or above 1.25 suggesting differences between reference and test treatment.

## Results

### Patient baseline characteristics

A total of 21 patients were enroled in the study, which finally comprised two cohorts (Table 1). Patients in each cohort had been pretreated for a variety of tumour types: all had received chemotherapy, and several had received other treatments including radiation therapy (*n*=11), hormones (*n*=1), or other agents (*n*=4).

### Treatment, dose reductions, and discontinuations

Because of DLT encountered in two out of six patients at the starting dose level, sunitinib was de-escalated to 25 mg per day. In a later stage the protocol was amended to expand cohorts with patients who had received no more than two previous chemotherapy regimens and had aspartate aminotransferase and alanine aminotransferase serum levels <2.5 × upper limit of normal (ULN; was <5 × ULN).

All patients received at least one full cycle of treatment, either at the sunitinib 37.5 mg per day (cohort 1) or at the sunitinib 25 mg per day (cohort 2) dose level. Those in cohort 1 received a median of six cycles of both sunitinib and irinotecan, whereas patients in cohort 2 received a median of 3 cycles of each agent. The majority of discontinuations from the study were due to lack of efficacy (*n*=8 in cohort 1; *n*=6 in cohort 2). Discontinuation due to adverse events during cycles 1 and 2 occurred in one patient, who experienced grade 3 febrile neutropenia and grade 5 pneumococcal sepsis.

During cycles 1 and 2, dose delay of sunitinib for ⩾1 week was required in three patients in cohort 1 and was not required in cohort 2; delays were required for irinotecan in four and one patients in cohorts 1 and 2, respectively.

Dose reductions of sunitinib occurred in three patients in cohort 1 during cycles 1 and 2, and were not required in cohort 2; no patients required more than one sunitinib dose reduction. For irinotecan, six patients in cohort 1 and one patient in cohort 2 required dose reduction during the first two cycles of treatment.

### DLTs, safety, and tolerability

In the sunitinib 37.5 mg per day group, including the expansion of this cohort, a total of four patients experienced a grade 3 or 4 adverse event during the first 2 cycles of treatment that was categorised as DLT: two patients had grade 4 neutropenia, one patient had grade 5 pneumococcal sepsis, and one patient had grade 3 fatigue. At the de-escalated dose of sunitinib 25 mg per day in cohort 2, no DLTs were observed, and the MTD was therefore defined as this dose level.

One patient in the sunitinib 37.5 mg per day group discontinued from the study because of a treatment-related adverse event (reactivation of hepatitis B). There were four deaths during the study, of which three were due to adverse events that were considered to be related to treatment with irinotecan and sunitinib: neurological disorder (*n*=1; cohort 2), septic shock (*n*=1; cohort 1), and pneumococcal infection and neutropenic fever (*n*=1; cohort 1).

The most frequent adverse events of any cause during cycles 1 and 2 (all seen in ⩾60% of patients overall) were vomiting, diarrhoea, and neutropenia. Events occurring in >25% of patients overall during cycles 1 and 2 are shown in Table 2.

Adverse events in patients receiving sunitinib 25 mg per day with irinotecan were predominantly grade 1 or 2 and nonhaematological in nature. In these patients, the most common grade ⩾3 all-causality adverse events in cycles 1 and 2 were neutropenia (*n*=2) and vomiting (*n*=2; Table 3). In patients receiving sunitinib 37.5 mg per day with irinotecan, the most common grade ⩾3 adverse events during the first two cycles were haematological: neutropenia (*n*=8) and leukopenia (*n*=6; Table 3).

### Efficacy

**Figure 1:**
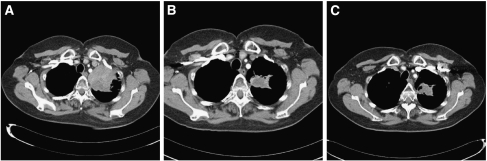
Partial response in a patient with inoperable non-small-cell lung cancer receiving sunitinib 37.5 mg per day and irinotecan. CT scans are shown at baseline (**A**), after 7 weeks (**B**) and 6 months (**C**) on treatment.

**Figure 2:**
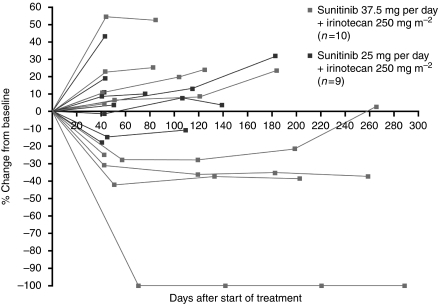
Percentage change from baseline in sum of target lesions (mm) in patients assessed for treatment response.

The majority of patients (10 out of 11 patients in the sunitinib 37.5 mg per day group and all 10 patients in the 25 mg per day group) had measurable disease at baseline, and were available for assessment of tumour response (Table 4). Out of 10 evaluable patients, 3 (30%) in cohort 1 had a confirmed partial response: one patient with submandibular cancer, one patient with oropharyngeal cancer who experienced complete regression of the target lesion in the presence of persisting non-target lesions, and one patient with relapsed NSCLC. Figure 1 shows durable tumour shrinkage in the patient with NSCLC. A further two patients in cohort 1 experienced stable disease for ⩾12 weeks (one patient each with CRC and leiomyosarcoma). In cohort 2, two patients (one patient each with prostate cancer and cervical cancer) had a best response of stable disease for ⩾12 weeks. Tumour response assessments (percentage change from baseline in sum of target lesions) are shown for individual patients in Figure 2.

### Pharmacokinetics

A summary of paired observations with respect to each analyte is shown in Table 5. Data for the 25 mg per day and 37.5 mg per day dose levels of sunitinib were reported in combination after dose correction to the MTD (25 mg for sunitinib and 250 mg m^−2^ for irinotecan). Where doses differed within paired observations, dose corrections to the intended dose were also performed. Based on this analysis, changes in pharmacokinetic values for sunitinib, total drug (sunitinib+SU012662), irinotecan, and SN-38 were within the range of variability of the data. SU012662 presented higher geometric mean ratios, which was mostly due to an apparent lower plasma exposure with the first dose (day −7; sunitinib alone).

For patients in both cohorts combined, the geometric mean ratios (sunitinib+irinotecan relative to sunitinib alone and sunitinib+irinotecan relative to irinotecan alone) of the pharmacokinetic parameter that related to maximum and total plasma exposure (i.e., *C*_max_, AUC_0–last_ and AUC_0–∞_) were calculated. The geometric mean ratios (sunitinib+irinotecan relative to sunitinib alone) of *C*_max_ and AUC_0–last_, for both cohorts combined were 0.82 and 0.88, respectively. Similarly, the geometric mean ratios for total drug were 0.90 and 0.95 for *C*_max_ and AUC_0–last_, respectively, suggesting that the pharmacokinetics of sunitinib when coadministered with irinotecan did not appear to change as compared with when it was administered alone.

The higher geometric mean ratios and apparent lower plasma exposure of SU012662 could potentially have been related to low-capacity tight/target tissue binding sites, which would be only present after the first dose at very low concentrations with limited sampling. Therefore, the increase in the geometric mean ratios for SU012662 was potentially caused by the limited sampling scheme and not because of a decrease in the elimination of the metabolite when sunitinib was coadministered with irinotecan as compared with its administration alone.

The geometric mean ratios (sunitinib+irinotecan relative to irinotecan alone) of *C*_max_, AUC_0–last_ and AUC_0–∞_ for both cohorts combined were 1.21, 1.12, and 1.13, respectively. Similarly, the geometric mean ratios of *C*_max_ and AUC_0–last_ for SN-38 were 1.13 and 1.20, respectively. Therefore, based on these data, coadministration of sunitinib with irinotecan did not appear to affect the pharmacokinetics of irinotecan or its active metabolite SN-38.

## Discussion

The current standard of care for treatment of patients with advanced CRC is FOLFOX or FOLFIRI with bevacizumab. Based on the rationale for combining a tyrosine kinase inhibitor of VEGFRs with chemotherapy, the combination of sunitinib plus irinotecan might offer efficacy in this patient category. In our dose-finding study, evidence is presented for objective responses in patients treated with the higher dose of sunitinib 37.5 mg per day in combination with irinotecan. At this dose, one patient with NSCLC and two with tumours in the head-and-neck region achieved a partial response. A further two patients (one patient each with CRC and leiomyosarcoma) maintained stable disease for ⩾12 weeks. It may be that durable stable disease, rather than objective response, is the main efficacy benefit when sunitinib is added to chemotherapy. The lower-dose regimen (sunitinib 25 mg per day with irinotecan), however, did not result in clinically meaningful responses.

Our dose-finding study indicated that in combination with irinotecan, the sunitinib dose of 37.5 mg per day was not well tolerated, with DLTs (including grade 4 neutropenia, grade 5 pneumococcal sepsis, and grade 3 fatigue) reported in four patients. Therefore, the MTD was determined to be sunitinib 25 mg per day, a dose level which was not associated with any DLTs. Indeed, the tolerability and safety results from this study in pretreated patients show that sunitinib 25 mg per day (days 1–14) with irinotecan 250 mg m^−2^ (day 1) in a 21-day cycle has a manageable safety profile. Most adverse events were mild–moderate at this dose, and haematological events, especially neutropenia and leukopenia, occurred at a lower frequency as compared with the higher dose.

Toxicities due to irinotecan normally include neutropenia, diarrhoea, and vomiting (Fuchs *et al*, 2003). In our study, these were among the most common all-causality adverse events, occurring in more than half of patients during cycles 1 or 2. Irinotecan/SN-38 toxicity is known to be affected by genetic and physiological variation in uridine-diphosphoglucuronosyl transferase 1A1 (UGT1A1) enzyme activity. Genotyping of UGT1A1 to permit potential reduction of drug dosing in patients with the UGT1A1^*^28 polymorphism is increasingly being carried out to avoid severe toxicities (Deeken *et al*, 2008; Funke *et al*, 2008; Rouits *et al*, 2008). Although 250 mg m^−2^ is a relatively low irinotecan dose, two patients in this study who developed grade 4 neutropenia within the first 2 weeks of cycle 1 were found to have decreased metabolism of SN-38 to its metabolite SN-38-glucuronide because of a UGT1A1^*^1/^*^28 and UGT1A1^*^28/^*^28 genotype, respectively.

The pharmacokinetic parameter values for sunitinib, SU012662, total drug (sunitinib + SU012662), irinotecan, and its active metabolite were consistent with data previously reported for sunitinib (Faivre *et al*, 2006; Britten *et al*, 2008), as well as for irinotecan (Rea *et al*, 2005; Camptosar, 2008). For patients in both cohorts combined, the geometric mean ratios (sunitinib+irinotecan relative to sunitinib alone, and sunitinib+irinotecan relative to irinotecan alone) of the pharmacokinetic parameters that related to maximum and total plasma exposure (i.e. *C*_max_, AUC_0–last,_ and AUC_0–∞_, respectively) were calculated. It could be concluded that no significant drug–drug interaction was found between sunitinib and irinotecan.

In conclusion, the results of this study suggest that no clinically relevant pharmacokinetic interactions occur when sunitinib is administered on days 1–14 of a 21-day cycle with irinotecan given on day 1. The MTD was defined as sunitinib 25 mg per day (days 1–14) and irinotecan 250 mg m^−2^ on day 1; this combination was reasonably well tolerated, but did not show preliminary antitumour activity. Evidence of activity was observed at the higher 37.5 mg per day dose, but this exceeded the MTD. Therefore, this particular combination will not be pursued for further studies in unselected patient populations. UGT1A1 genotyping was not routinely performed in our study and may have been useful to identify patients with the UGT1A1^*^28 polymorphism who required irinotecan dose reduction. As a VEGFR tyrosine kinase inhibitor combined with chemotherapy did not appear to have synergistic antitumour activity in our study (as also observed in most phase III, randomised trials, in contrast to an anti-VEGF monoclonal antibody plus chemotherapy; Los *et al*, 2007), we do not recommend further phase II or III studies with this combination.

## Figures and Tables

**Table 1:** Patient baseline characteristics

	**Cohort 1 sunitinib 37.5 mg per day+irinotecan 250 mg m^−2^ (*n*=11)**	**Cohort 2 sunitinib 25 mg per day+irinotecan 250 mg m^−2^ (*n*=10)**
Median (range) age, years	50 (42–62)	51 (32–67)
Male/female, *n* (%)	8 (73)/3 (27)	4 (40)/6 (60)
		
*ECOG performance status, n* (%)		
0	6 (55)	3 (30)
1	5 (45)	6 (60)
2	0	1 (10)
		
*Tumour types, n*		
Colorectal carcinoma	1	3
Non-small-cell lung carcinoma	2	1
Cervical carcinoma	1	2
Head-and-neck tumour	2	0
Breast carcinoma	0	1
Gall bladder carcinoma	1	0
Gastric carcinoma	1	0
Mesothelioma	1	1
Osteosarcoma	1	0
Prostate carcinoma	0	1
Soft tissue sarcoma	1	0
Mediastinal carcinoid	0	1

Abbreviation: ECOG, eastern cooperative oncology group.

**Table 2:** Adverse events (AEs) occurring in >25% of patients during cycles 1 and 2 and during all cycles (all grades, all causalities)

	**Cohort 1 sunitinib 37.5 mg per day+irinotecan 250 mg m^−2^** **(*n*=11)**		**Cohort 2 sunitinib 25 mg per day+irinotecan 250 mg m^−2^** **(*n*=10)**	
**AE, *n* (%)**	**Cycles 1 and 2**	**All cycles**	**Cycles 1 and 2**	**All cycles**
Neutropenia	11 (100)	11 (100)	6 (60)	6 (60)
Nausea	8 (73)	8 (73)	4 (40)	9 (90)
Vomiting	6 (55)	8 (73)	8 (80)	9 (90)
Leukopenia	9 (82)	10 (91)	4 (40)	5 (50)
Diarrhoea	9 (82)	9 (82)	6 (60)	6 (60)
Anorexia	7 (64)	8 (73)	2 (20)	4 (40)
Asthenia	5 (45)	6 (55)	4 (40)	5 (50)
Abdominal pain	6 (55)	6 (55)	4 (40)	5 (50)
Alopecia	5 (45)	7 (64)	4 (40)	4 (40)
Fatigue	4 (36)	4 (36)	3 (30)	5 (50)
Headache	3 (27)	4 (36)	4 (40)	5 (50)
Dyspnoea	3 (27)	3 (27)	4 (40)	6 (60)
Anaemia	3 (27)	6 (55)	3 (30)	3 (30)
Mucosal inflammation	5 (45)	5 (45)	1 (10)	2 (20)
Thrombocytopenia	3 (27)	4 (36)	2 (20)	2 (20)

**Table 3:** Most frequent (occurred in ⩾2 patients) adverse events (AEs), grade ⩾3, all causalities

	**Cohort 1 sunitinib 37.5 mg per day+irinotecan 250 mg m^−2^** **(*n*=11)**		**Cohort 2 sunitinib 25 mg per day+irinotecan 250 mg m^−2^** **(*n*=10)**	
**AE, *n* (%)**	**Cycles 1 and 2**	**All cycles**	**Cycles 1 and 2**	**All cycles**
*Haematological*				
Neutropenia	8 (73)	8 (73)	2 (20)	3 (30)
Leukopenia	6 (55)	6 (55)	1 (10)	1 (10)
Anaemia	1 (9)	2 (18)	1 (10)	1 (10)
Thrombocytopenia	0	1 (9)	1 (10)	1 (10)
				
*Nonhaematological*				
Asthenia	2 (18)	3 (27)	1 (10)	3 (30)
Vomiting	1 (9)	2 (18)	2 (20)	3 (30)
Fatigue	1 (9)	1 (9)	1 (10)	2 (20)
Nausea	1 (9)	1 (9)	0	1 (10)
Abdominal pain	0	1 (9)	1 (10)	1 (10)
Gamma glutamyl transferase increase	1 (9)	2 (18)	0	0

**Table 4:** Patients' best tumour response to treatment according to RECIST 1.0

**Patients, *n* (%)**	**Cohort 1 sunitinib 37.5 mg per day+irinotecan 250 mg m^−2^** **(*n*=10)**	**Cohort 2 sunitinib 25 mg per day+irinotecan 250 mg m^−2^** **(*n*=10)**
Partial response	3 (30)	0
Stable disease ⩾12 weeks	2 (20)	2 (20)
Progressive disease	3 (30)	4 (40)
Not evaluable	2 (20)	4 (40)

Abbreviation: RECIST 1.0=Response Evaluation Criteria in Solid Tumors version 1.0.

**Table 5:** Summary of pharmacokinetic values (arithmetic mean (coefficient of variation)) for sunitinib, SU012662, sunitinib+SU012662, irinotecan, and its active metabolite SN-38 for all doses combined (paired observations only)

**Pharmacokinetic parameter**	**Sunitinib alone C0D–7, mean (CV%)**	**Irinotecan alone C1D1, mean (CV%)**	**Sunitinib+irinotecan C2D1, mean (CV%)**	**Geometric mean ratio (C2D1/C0D–7 or C2D1/C1D1)**
*Sunitinib (n*=*18)*^a^				
T_max_ (h)^b^	8 (4–24)	NA	8 (2–29)	
T_last_ (h)^c^	24.0	NA	24.0	
C_max_ (ng l^−1^)	16.3 (39.2)	NA	13.0 (31.8)	0.82
AUC_0–last_ (ng h ml^−1^)	266.5 (41.2)	NA	225.5 (29.5)	0.88
				
*SU012662 (n*=*18)*^a^				
T_max_ (h)^b^	7 (2–24)	NA	24 (1.8–29)	
T_last_ (h)^c^	24.0	NA	24.0	
C_max_ (ng ml^−1^)	2.4 (51.4)	NA	3.6 (32.5)	1.6
AUC_0–last_ (ng h ml^−1^)	38.6 (54.4)	NA	53.2 (30.9)	1.48
				
*Total drug (n*=*18)*^a^				
*T*_max_ (h)^b^	8 (4–24)	NA	9 (2–29)	
*T*_last_ (h)^c^	24.0	NA	24.0	
*C*_max_ (ng ml^−1^)	18.5 (40.3)	NA	16.2 (29.7)	0.90
AUC_0–last_ (ng h ml^−1^)	305.2 (42.1)	NA	278.2 (28.7)	0.95
				
*Irinotecan (n*=*18–20)*^c^				
*T*_max_ (h)^b^	NA	1 (1–1.7)	1 (0.9–1.7)	
*T*_last_ (h)^c^	NA	24.0	24.0	
*C*_max_ (*μ*g ml^−1^)	NA	2.9 (21.3)	3.6 (26.8)	1.21
AUC_0–last_ (*μ*g h ml^−1^)	NA	14.9 (27.7)	16.9 (33.3)	1.12
AUC_0–∞_ (*μ*g h ml^−1^)	NA	15.9 (29.3)	18.1 (34.0)	1.13
CL (l h^−1^)	NA	31.9 (30.3)	30.4 (28.8)	
t_1/2_ (h)	NA	6.3 (19.8)	6.6 (21.9)	
				
*SN-38 (n*=*18–20)*^c^				
T_max_ (h)^b^	NA	1.1 (1.0–4.2)	1.5 (0.9–4.1)	
T_last_ (h)^c^	NA	24.0	24.0	
C_max_ (*μ*g ml^−1^)	NA	0.03 (51.4)	0.04 (50.0)	1.13
AUC_0–last_ (*μ*g h ml^−1^)	NA	0.31 (61.7)	0.36 (52.8)	1.20

Abbreviations: AUC_0–∞_=area under the plasma concentration–time curve (AUC) from time zero to infinity; AUC_0–last_=AUC from time zero to the last quantifiable sampling time point; C0D–7=cycle 0 (screening) day –7; C1D1=cycle 1 day 1; C2D1=cycle 2 day 1; CL=clearance; C_max_=maximum plasma concentration; CV=coefficient of variation; NA=not applicable; t_1/2_=terminal elimination half-life; *T*_last_=time when last sample collected; *T*_max_=time to *C*_max_.

a Pharmacokinetic parameters estimated on C2D1 were corrected for carryover pre-dose concentrations where applicable.

b Median and range values presented for the parameter *T*_max_.

c Median value reported for *T*_last_.
